# Sink or Source: Alternative Roles of Glacier Foreland Meadow Soils in Methane Emission Is Regulated by Glacier Melting on the Tibetan Plateau

**DOI:** 10.3389/fmicb.2022.862242

**Published:** 2022-03-21

**Authors:** Tingting Xing, Pengfei Liu, Mukan Ji, Yongcui Deng, Keshao Liu, Wenqiang Wang, Yongqin Liu

**Affiliations:** ^1^State Key Laboratory of Tibetan Plateau Earth System, Resources and Environment (TPESRE), Institute of Tibetan Plateau Research, Chinese Academy of Sciences, Beijing, China; ^2^College of Resources and Environment, University of Chinese Academy of Sciences, Beijing, China; ^3^Center for the Pan-Third Pole Environment, Lanzhou University, Lanzhou, China; ^4^School of Geography, Nanjing Normal University, Nanjing, China; ^5^Jiangsu Center for Collaborative Innovation in Geographical Information Resource Development and Application, Nanjing, China

**Keywords:** methane flux, methanogens, methanotrophs, glacier foreland, Tibetan Plateau

## Abstract

Glacier foreland soils have long been considered as methane (CH_4_) sinks. However, they are flooded by glacial meltwater annually during the glacier melting season, altering their redox potential. The impacts of this annual flooding on CH_4_ emission dynamics and methane-cycling microorganisms are not well understood. Herein, we measured *in situ* methane flux in glacier foreland soils during the pre-melting and melting seasons on the Tibetan Plateau. In addition, high-throughput sequencing and qPCR were used to investigate the diversity, taxonomic composition, and the abundance of methanogenic archaea and methanotrophic bacteria. Our results showed that the methane flux ranged from −10.11 to 4.81 μg·m^−2^·h^−1^ in the pre-melting season, and increased to 7.48–22.57 μg·m^−2^·h^−1^ in the melting season. This indicates that glacier foreland soils change from a methane sink to a methane source under the impact of glacial meltwater. The extent of methane flux depends on methane production and oxidation conducted by methanogens and methanotrophs. Among all the environmental factors, pH (but not moisture) is dominant for methanogens, while both pH and moisture are not that strong for methanotrophs. The dominant methanotrophs were *Methylobacter* and *Methylocystis*, whereas the methanogens were dominated by methylotrophic *Methanomassiliicoccales* and hydrogenotrophic *Methanomicrobiales*. Their distributions were also affected by microtopography and environmental factor differences. This study reveals an alternative role of glacier foreland meadow soils as both methane sink and source, which is regulated by the annual glacial melt. This suggests enhanced glacial retreat may positively feedback global warming by increasing methane emission in glacier foreland soils in the context of climate change.

## Introduction

Methane (CH_4_) is the second most important greenhouse gas in the atmosphere, with 28 times the global warming potential of CO_2_ on the centennial-scale (IPCC, 2013). Methanogens and methanotrophs are responsible for methane-cycling in the natural environment, and their relative activities determine the global methane dynamics (Le Mer and Roger, 2001), while anaerobic methanotrophs, coupling methane oxidation to the reduction of nitrate, nitrite, iron, manganese, and sulfate, also play a role in the methane cycle (Knittel and Boetius, 2009; Ettwig et al., 2010, 2016; Haroon et al., 2013; Hu et al., 2014; Leu et al., 2020; Guerrero-Cruz et al., 2021). Methane flux in glacier foreland soils has attracted increased attention due to the rapid retreat of mountain glaciers under global warming (Bárcena et al., 2010). Methane dynamics in the natural environment depend on nutrient availability (Le Mer and Roger, 2001), vegetation coverage (Adachi et al., 2006), terrain topography (Wei et al., 2015b), soil moisture content (Coles and Yavitt, 2002; Wei et al., 2015a), and temperatures (Avery et al., 2003; Metje and Frenzel, 2005; Bárcena et al., 2010). Global warming has greatly enhanced glacier retreat, which accelerates the expansion of glacier foreland. Thus, the methane dynamics at glacier foreland soils can enhance or mitigate the impact of climate change.

Glacier foreland soils are typically regarded as a sink for methane, and most previous studies on methane-cycling microorganisms have focused on the community succession of methanotrophs (Nauer et al., 2012; Chiri et al., 2015, 2017; Mateos-Rivera et al., 2018). For example, a previous study measured the abundance of methanotrophs and revealed a stable CH_4_ uptake (−0.082 to −2.2 mg CH_4_ m^−2^ d^−1^) during the snow-free season in Damma and Griessfirm glacier forefields in Switzerland (Chiri et al., 2015). In contrast, a net CH_4_ production has been reported for some glacier foreland (e.g., the Swiss Alps glacier foreland; Bárcena et al., 2010; Nauer et al., 2012). These results suggest that glacier foreland soil may also serve as a potential role of methane sources. However, the driving factors that regulate the methane flux in glacier foreland are largely unknown.

Accelerated glacier retreat has occurred on the Tibetan Plateau since the early 20th Century (Yao et al., 2007), and the exposed barren soil is expected to develop into meadow within 80 years (Eichel, 2019). The alpine meadow soils on the Tibetan Plateau are considered an important sink for atmospheric CH_4_ (Wei et al., 2015b). In comparison, wetland and swamp meadows are major methane source regions, which could emit 0.56 ~ 1 Tg CH_4_ a^−1^ (Jin et al., 1999; Ding and Cai, 2007). Alpine meadow and swamp meadow are inter-convertible by the alternation of hydrological conditions. The enhanced glacial retreat can increase glacial meltwater discharge, which affects the hydrological processes in the glacier foreland regions (Yao et al., 2007; Bradley et al., 2014). During the melting seasons, a large amount of glacial meltwater flows into glacier foreland soils, transforming alpine meadow into swamp meadow, and reducing the oxygen availability, which favors methanogens over methanotrophs (Chen et al., 2008). This may transform the glacier foreland soils from a methane sink to a methane source, but the microbial process underlying this transformation is poorly understood.

The overall goals of this study were to: assess the impact of glacier melting on the dynamics of methane flux and identify the environmental and biological drivers of the methane dynamics. Based on the impact of glacier meltwater on the hydrological conditions in glacier foreland soils, we hypothesized that the methanogens would be more abundant than methanotrophs, and turn glacier foreland meadow into a methane source.

## Materials and Methods

### Foreland of the Longxiazailongba Glacier

**Figure 1:**
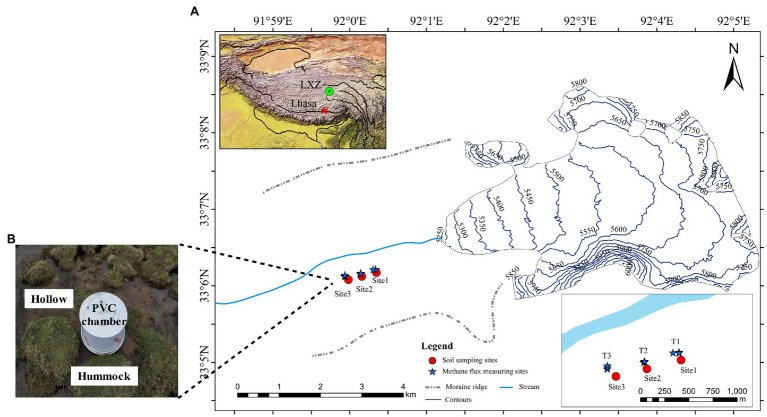
**(A)** Sites of soil sampling and *in situ* methane (CH_4_) flux measurement on the forefield of Longxiazailongba (LXZ) Glacier. The left inset map shows the locations of the glacier in the Tibetan Plateau, and the right down inset shows the detailed soil sampling sites and *in situ* CH_4_ measurement in the glacier foreland. Both the hollow and hummock soils were samples with three replicates for all three sites. The soil samples were collected in August 2017, and the *in situ* CH_4_ flux measuring was carried out in June (pre-melting season) and August (glacial melting season) in 2020. **(B)** During the melting season, the alpine meadow is transformed to the alpine wetland, and the hollow was submerged without vegetation, while hummocks were dominated by *Kobresia tibetica*. The CH_4_ flux was measured *in situ* using polyvinylchloride cylindrical (PVC) chambers method.

The Longxiazailongba (LXZ) Glacier is located in the middle of the Tanggula Mountain on the Tibetan Plateau, China (Figure 1). The glacier is 7 km in length, 19.3 km^2^ in area, 118 ± 10 m in thickness, and the highest altitude is 6,000 m above sea level (a.s.l.; Yao, 2014). The altitude of the glacier terminus is about 5,240 m a.s.l. The area near the terminus (about 0.5 km in length and 1.1 km in width) was moraine without any vegetation. Vegetation was developed at approximately 600 m from the glacier terminus, and meadows (dominated by *Kobresia tibetica*) were developed at approximately 1.5 km from the glacier terminus (Figure 1A).

The glacial meltwater enters the foreland from Mid-June, transforming the alpine meadow into a swamp meadow until October. Therefore, June is defined as pre-melting season, and July to October is defined as melting season in the present research. During the glacial melting season, the swamp meadow exhibits both hummock and hollow microtopography (Figure 1B), which are commonly observed in wetlands. Hummocks are typically located higher above the water table, and hollows are closer to the water table and may occasionally be flooded during glacial melt season (Belyea and Clymo, 1998). At the LXZ glacier foreland, hummock soils were covered by *K. tibetica*, while hollows were inundated with glacier meltwater and no vegetation was observed (Figure 1B).

### Soil Sampling

In August 2017, soil samples were collected at three sites at the glacier foreland during the melting season (Figure 1A). These meadows feature both hummock and hollow microtopography. At each site, soil samples were collected from three hollow or hummock microtopography as three independent replicates. In each microtopography, five surface soil samples (0–10 cm) were collected randomly using a sterile shovel and were then mixed thoroughly to form a composite hollow or hummock sample. The fresh soil samples were stored in Whirl-Pak bags at approximately 4°C in a portable refrigerator and delivered to the laboratory within 48 h. In the laboratory, soil samples were frozen at −80°C for further analysis.

### Soil Characteristics Measurement

Moisture was measured by using the gravimetric method. Approximately 10 g 2 mm sieved fresh soil was weighted, and oven-dried at 105°C until no further mass loss was observed and then reweighted. The moisture content is expressed as the mass of water per mass of dry soil. The pH values were measured after mixing wet soil with distilled water at the soil-to-water ratio of 1:5 (g/g). Soil organic matter (OM) was determined by the external heating-potassium dichromate volumetric method with air-dried soils (Ji, 2005). The total nitrogen content (TN) was determined using the Kjeldahl method (Bremner, 1960). Soil ammonium nitrogen (NH+ 4) and nitrate-nitrogen (NO-3) were extracted from wet soils with 2 M KCl (soil/solution, 1:5) using Smartchem200 Discrete Auto Analyzer (Alliance, France). The available phosphorus contents (AP) were determined using the acid digestion method (Kuo, 1996).

### DNA Extraction and Quantitative PCR

Genomic DNA was extracted from 0.5 g soil using Fast DNA®SPIN Kit for Soil (MP Biomedicals, Santa, CA, United States). The Quality and quantity of the extracted DNA were measured using a NanoDrop 2000 Spectrophotometer (Thermo-Scientific). In order to minimize the potential inhibitory effects of co-extracted substrates from soil (e.g., humic acid), soil DNA was diluted 10 times for qPCR. The m*crA* gene encodes the α-subunit of methanogenic methyl coenzyme M reductase, and the *pmoA* gene encodes the α-subunit of methane monooxygenase, which is used for the initial conversion of CH_4_ to methanol. The copy numbers of *mcrA* and *pmoA* genes were quantified using qPCR with primer mals-mod-F (5′-GGYGGTGTMGGDTTCACMCARTA-3′)/mcrA-rev-R (5′-CGTTCATBGCGTAGTTVGGRTAGT-3′; Angel et al., 2012) and A189f (5′-GGNGACTGGGACTTCTGG-3′)/mb661r (5′-CCGGMGCAACGTCYTTACC-3′; Kolb et al., 2003), respectively. Quantitative PCR amplification was performed on a CFX-96 Optical Real-Time PCR System (Bio-Rad Inc. Hercules, CA, United States). The 20 μl reaction mixture contained 2 μl of the DNA template, 10 μl of TB GreenTM Premix EX TaqTM II (TaKaRa), 0.4 μl of each forward and reverse primer (20 μM), and 7.2 μl ddH_2_O. qPCR conditions for *mcrA* is as follows: initial denaturation (94°C, 6 min), followed by 50 cycles of denaturation (94°C, 25 s), annealing (65.5°C, 20 s), and elongation (72°C, 45 s). While qPCR condition for *pmoA* is as follows: initial denaturation at 95°C for 30 s, followed by 40 cycles of 5 s at 95°C, 30s at 54°C, 40s at 72°C, and 30s at 80°C. A melting curve analysis was conducted to confirm the specificity of the PCR products.

### *In situ* CH_4_ Flux Measurement

Methane flux was measured *in situ* in June (pre-melting season) and August (melting season) 2020. Methane flux was measured using polyvinylchloride cylindrical (PVC) tubes at site 1 to site 3 (Figure 1). Tubes consist of two parts, a PVC base (25 cm in diameter and 10 cm in height) installed permanently into the soil of about 5 cm, and a cylindrical box (without bottom, 25 cm in diameter and 30 cm in height; Figure 1B). In the field, six chambers were placed simultaneously, with three chambers placed on hollow soils and the other three on hummock soils. Chambers were closed for 20 min before the gas was collected. The gas was collected every 15 min using a plastic syringe (100 ml) and stored in a sterile gas package (Haide, Dalian).

The gas samples were analyzed within 48 h using a gas chromatograph (Agilent GC-7890B, United States) at the Naqu Ecological and Environmental Observation and Research Station, China (31°17’N, 92°06′E; 4,501 m a.s.l.). The gas chromatograph was equipped with a flame ionization detector (FID) and electron capture detector (ECD), using N_2_ as the carrier gas to remove O_2_ and water vapor (Chen et al., 2017).

The CH_4_ flux was calculated as the following (Wang and Wang, 2003):


$$F=\frac{M}{V_{0}}\frac{P}{P_{0}}\frac{273}{T+273}\frac{△C}{△t}H.$$

Where, *F* is the methane flux (mg·m^−2^·h^−1^), *M* is the molecular mass of methane (16.12 g/mol), *V_0_* is the gas molecular mass (22.41 L/mol), *P* is the atmospheric pressure at the sampling site, *P_0_* is standard atmospheric pressure (1013.25 mbar), $\frac{△C}{△t}$ is the slope of the linear regression for the methane concentration gradient through time (m^3^·m^−3^·h^−1^), and *H* is the chamber height above the soil (m).

### High-Throughput Sequencing and Data Analysis

The compositions of methanogenic and methanotrophic communities were evaluated using high-throughput sequencing targeting the *mcrA* and *pmoA* genes, respectively. The primer pairs for *mcrA* and *pmoA* genes were mlas-mod-F/mcrA-rev-R and A189f/mb661r, respectively (Costello and Lidstrom, 1999; Angel et al., 2012). PCR amplification was performed with primers with sample-specific barcodes. Around 35 cycles were used to amplify both the *mcrA* gene and *pmoA* gene PCR products. The PCR products of each sample were purified and quantified using a Qubit instrument (Life Technologies). The PCR products were pooled in an equimolar concentration, and sequenced on an Illumina MiSeq system using 2 × 300 cycle combination mode at Shanghai Meiji Biotechnology Co., Ltd.

For the *mcrA* and *pmoA* gene sequence analyses, the paired-end sequences were merged and quality checked. Candidate OTUs sequences of *mcrA* and *pmoA* genes were obtained using the “unoise3” command in Usearch v11.0.667 (Edgar, 2013). Chimeras were removed during the clustering process. These candidate OTUs sequences were uploaded to FrameBot (Wang et al., 2013) to check the frameshift and delete frameshift sequences. In addition, the candidate OTUs sequences were imported into ARB (Ludwig et al., 2004) to calculate the distance matrix based on amino acid sequences. Using cluster command in Mothur (Schloss et al., 2009), the final *mcrA* and *pmoA* OTUs at approximate species-level were assigned with amino acid dissimilarity levels of 0.11 (Yang et al., 2014) and 0.07 (Lüke and Frenzel, 2011), respectively. Meantime, we double-checked the number of new *mcrA* OTUs based on the distance level of 0.11 amino acids sequences, which was equal to the number obtained based on 0.84 nucleotide distance (Yang et al., 2014). Finally, based on the representative sequences obtained above, the OTU table was generated using the “otutab” command in Usearch.

To establish the phylogenetic trees for methanogens and methanotrophs, representative OTUs sequences and the reference sequences were first aligned using mafft v7.464 (Rozewicki et al., 2019). AliView (Larsson, 2014) was used to check and realign the aligned sequences based on the length of sequences that automatically trimmed by trimAI (Capella-Gutiérrez et al., 2009). FastTree v2.1 (Price et al., 2010) was used to generate the approximate maximum likelihood tree. Finally, these tree files were visualized in iTOL (Letunic and Bork, 2016).^1^ Meanwhile, the taxonomic information of the representative OTUs was assigned according to the relationship between the representative OTUs and the reference sequences.

### Statistical Analyses

The Kruskal–Wallis test was used to identify the significant differences in soil physicochemical properties, methane-cycling microorganisms, and methane fluxes among different microtopography using the software of PAST v3.0 (Hammer et al., 2001). Alpha diversity of Chao1 richness and Shannon indexes were processed with *vegan* package v2.5-7 (Dixon, 2003). Spearman correlations were used to determine the relationships of methane flux and methane cycling communities with soil properties. The heatmap was performed with the package of “*heatmap*” (Kolde, 2013) in R 4.0.4.

## Results

### Soil Physiochemical Properties

The physicochemical properties of glacier foreland soils are shown in Supplementary Table S1. All soils were neutral to slight alkaline with pH values ranging from 6.94 to 8.14. The air temperature was 10.43 ± 5.06°C and 10.96 ± 4.71°C when methane flux was measured in June and August, and no significant difference was detected (Kruskal–Wallis test, *p* = 0.282). The pH values of hollow soils were significantly higher than those of hummock soils (Kruskal–Wallis test, *p* < 0.001). In contrast, the available phosphorous (AP) was significantly higher in hummock soils than in hollow soils (*p* = 0.013). Other soil properties, including soil water content, values of total nitrogen (TN), ammonium (NH+ 4), nitrate (NO-3), and organic matter (OM) had no significant differences between hollow and hummock samples.

### The Abundances of Methane-Cycling Microorganisms and Methane Flux

**Figure 2:**
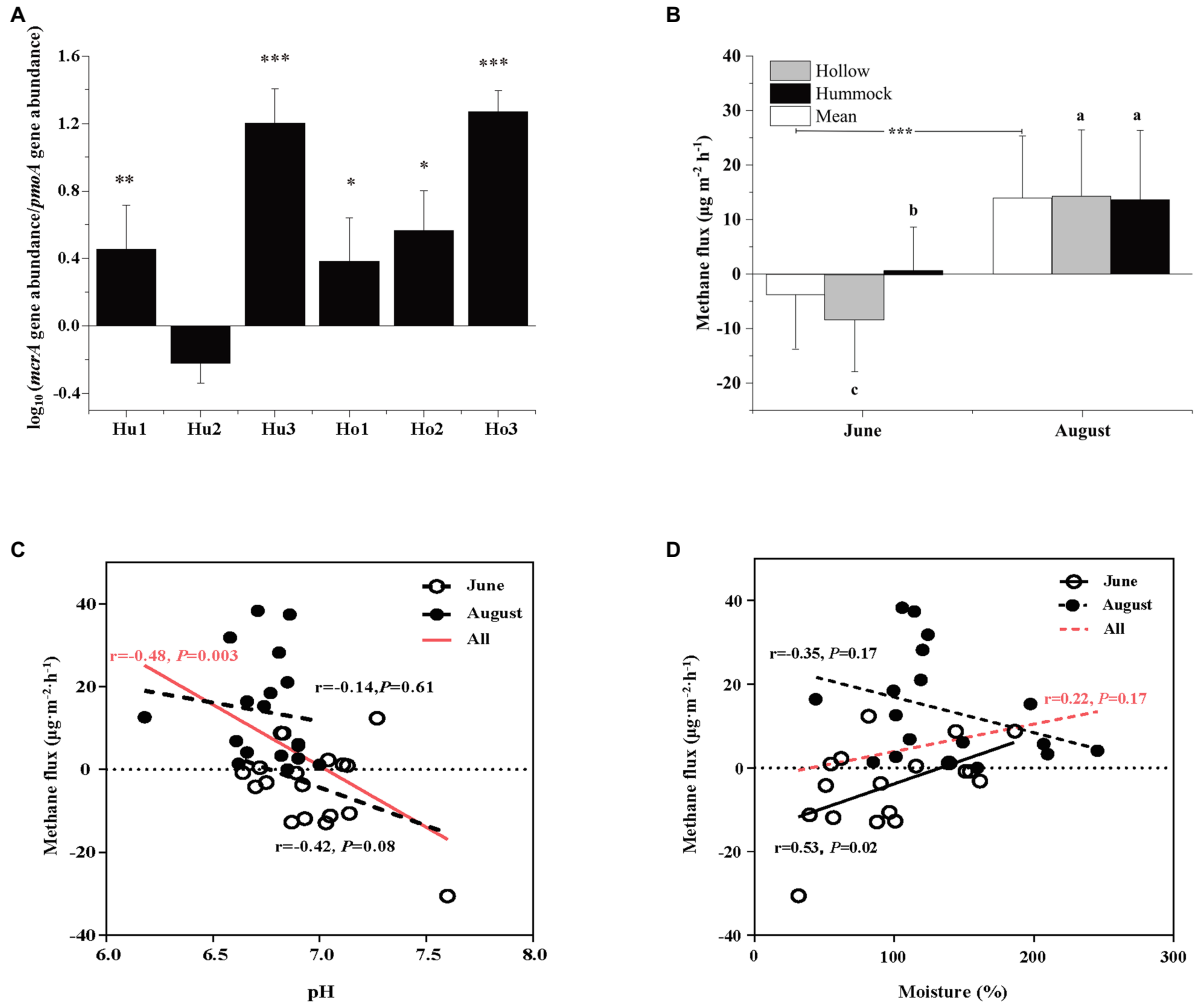
**(A)** Comparison of Log-transformed *mcrA*-to-*pmoA* gene copy numbers. Asterisks indicate the significant higher abundance of *mcrA* gene copy numbers (i.e., ratio > 1, ^***^p < 0.001, ^**^p < 0.01, and ^*^p < 0.05). **(B)** The mean methane flux in June and August (white), and the methane flux in hollow (gray), and hummock soils (black) in June (pre-melting season) and August (melting season). Asterisks indicate significant differences between June and August measurements. Significant differences (*p* < 0.05) by microtopography are indicated by different letters. **(C)** The relationships of methane flux in June (empty circle) and August (solid circle) with soil pH. **(D)** The relationships of methane fluxes in June (empty circle) and August (solid circle) with soil water content. The red lines in **(C,D)** indicate the relationship between methane fluxes (both June and August) with soil pH and soil water content. Error bars in **(A,B)** represent SD (*n* = 3). The relationships are fitted by linear model and significant differences (*p* < 0.05) were displayed by solid lines, whereas dotted lines indicate non-significant relationships (*p* > 0.05).

**Figure 3:**
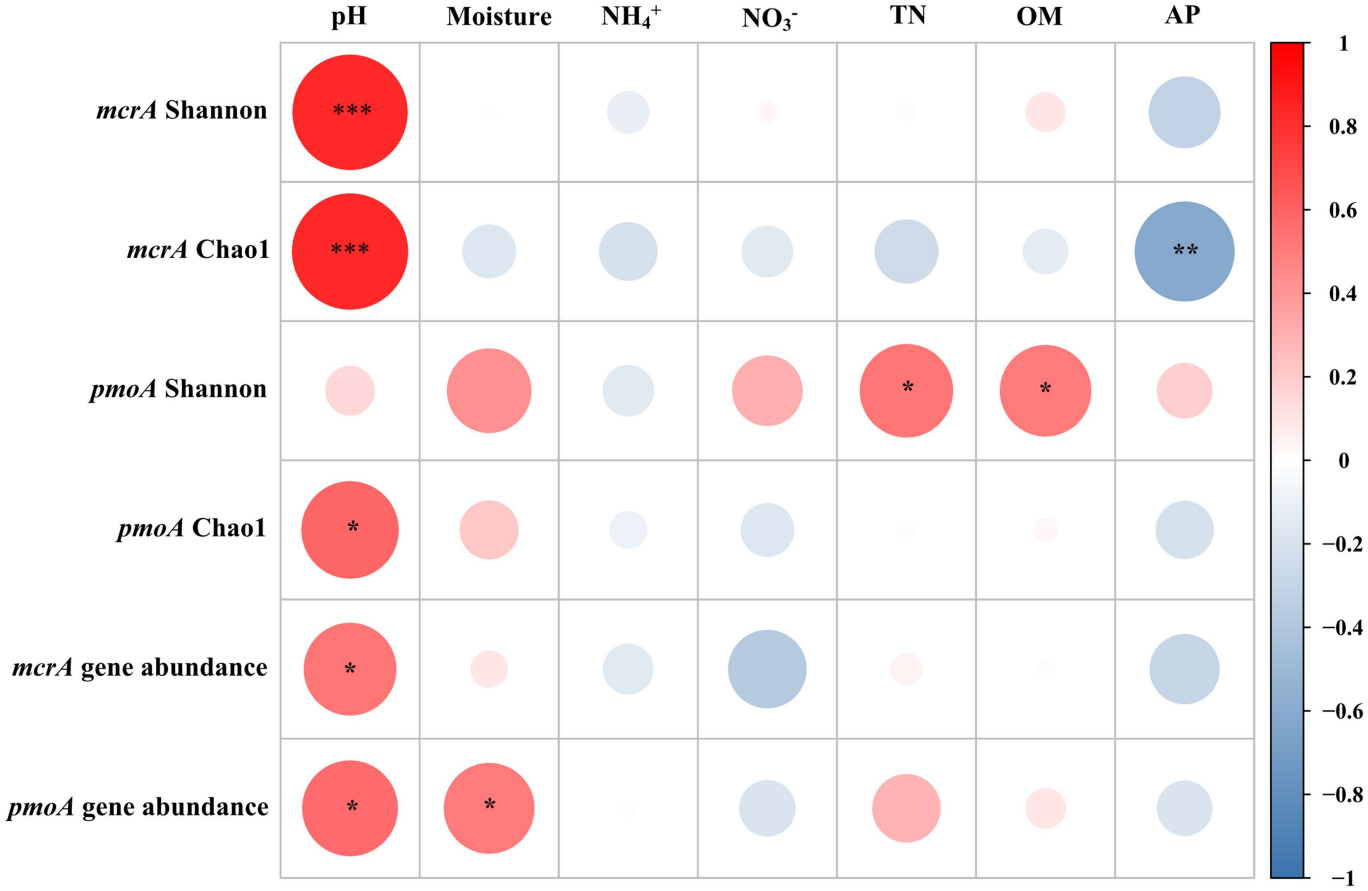
Correlation analyses of diversity indices and abundances of methanogens (*mcrA*) and methanotrophs (*pmoA*), and *mcrA* and *pmoA* gene abundances with soil properties. Asterisks represent statistically significant relationships (^***^p < 0.001, ^**^p < 0.01, and ^*^p < 0.05). The color code depicted the *r-value* of Spearman correlations.

The abundance of methanogens was inferred by the *mcrA* gene copy number, which ranged from 1.19 × 10^6^ to 5.17 × 10^8^ copies g^−1^ dry soil. The abundance of methanotrophs was inferred using the *pmoA* gene copy number, which ranged from 4.76 × 10^5^ to 1.09 × 10^8^ copies g^−1^ dry soil (Supplementary Table S2). Except for the hummock soils in site 2, the *mcrA*-to-*pmoA* gene copy number ratios were consistently greater than 1 (*z*-test, all *p* < 0.05; Figure 2A). Correlation analysis indicated that both *mcrA* and *pmoA* gene abundance were significantly and positively correlated to soil pH (Figure 3). In addition, *pmoA* gene abundance was also significantly and positively correlated to soil water content, while no significant correlation was observed between soil water content and *mcrA* gene abundance (Figure 3).

To evaluate the impact of glacier meltwater on methane dynamics in glacier foreland soils, methane fluxes during the pre-melting season (June) and glacier melting season (August) were measured. In the pre-melting season, the methane flux was −3.76 ± 9.84 μg·m^−2^·h^−1^, and significantly increased to 13.95 ± 12.44 μg·m^−2^·h^−1^ in the glacier melting season (Kruskal–Wallis test, *p* < 0.001; Figure 2B). Methane flux variations were also observed by the microtopographic effects, the methane flux was −8.29 ± 9.44 μg·m^−2^·h^−1^ in hollow soils in the pre-melting season, which was significantly higher than that in hummock soils (0.78 ± 7.97 μg·m^−2^·h^−1^, *p* = 0.027). However, such variation diminished during the glacier melting season, i.e., the methane flux in hollow soils was 14.27 ± 12.15 μg·m^−2^·h^−1^, which was no longer significantly different from that in hummock soils (13.64 ± 12.71 μg·m^−2^·h^−1^, *p* = 0.964). There is a negative and statistically significant correlation between the methane flux and soil pH (*r* = −0.48, *p* = 0.003; Figure 2C). However, the correlation between methane flux and pH was insignificant when the methane flux in the pre-melting season and melting season were investigated separately (*p* = 0.08 and 0.75, respectively). Furthermore, the methane flux significantly correlated with soil moisture in the pre-melting season (*p* = 0.02), but not in the melting season (*p* = 0.17; Figure 2D).

### The Diversity of Methane-Cycling Microorganisms in Glacier Foreland Soils

For methanogens, the Chao1 richness and Shannon indexes ranged from 5.0 to 13.0, and 1.3 to 3.1, respectively. Both indexes were significantly higher in hollow soil samples than those in hummock soil samples (Kruskal–Wallis test, all *p* < 0.001; Supplementary Figures S1A,C). The Chao1 richness and Shannon indexes of the methanotrophs ranged from 4 to 11, and 0.58 to 2.27, respectively, and no significant differences in both alpha diversity indexes were observed between the hollow and hummock soils (*p* = 0.19 and *p* = 0.44, respectively; Supplementary Figures S1B,D).

Spearman correlation analyses demonstrated that both Chao1 richness and Shannon indexes of the methanogens were significantly and positively correlated with soil pH (*r* = 0.82, *p* < 0.001; Figure 3). The Chao1 richness index of the methanotrophs was positively correlated with pH (*r* = 0.58, *p* = 0.02), and the Shannon index was positively correlated with OM (*r* = 0.54, *p* = 0.02) and TN (*r* = 0.51, *p* = 0.03).

### Methanogenic and Methanotrophic Community Compositions

**Figure 4:**
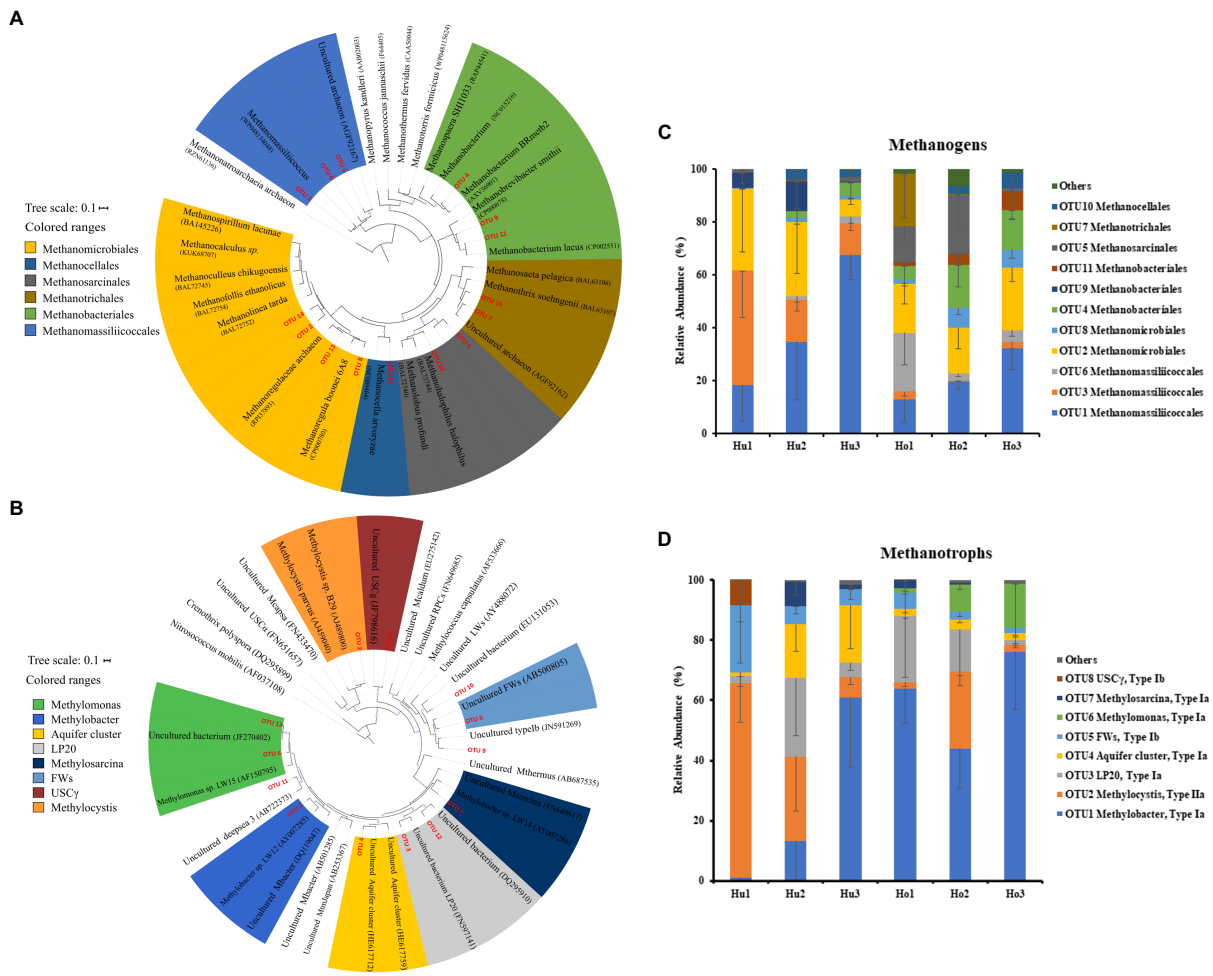
Phylogenetic analysis of *mcrA* **(A)** and *pmoA* **(B)** genes, and the relative abundance of *mcrA* **(C)** and *pmoA* OTUs **(D)** in hollow (Ho 1 to Ho 3) and hummock soils (Hu 1 to Hu 3). Error bars indicate SD (*n* = 3).

**Figure 5:**
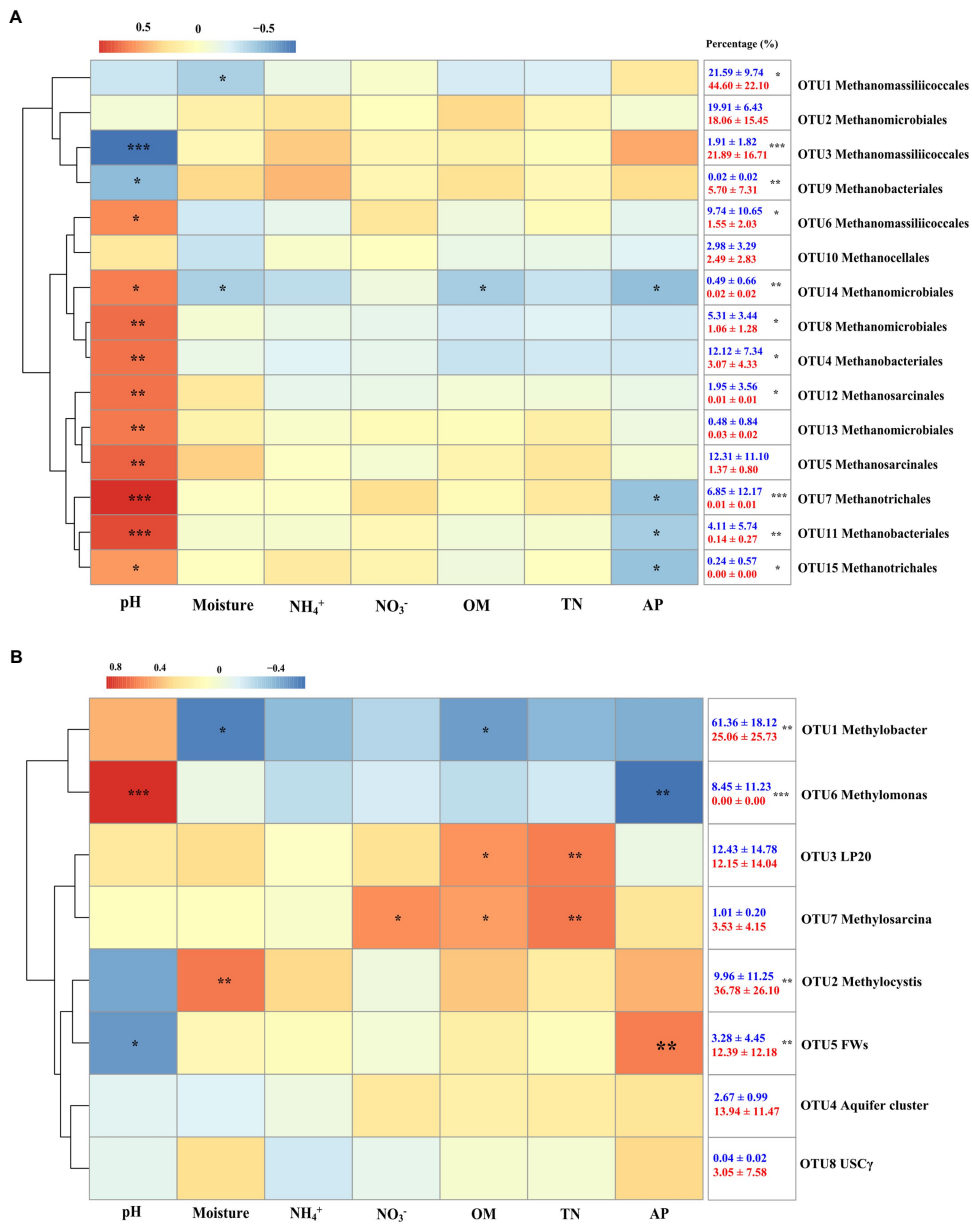
Heatmap of Spearman correlations of the relative abundance of methanogens **(A)** and methanotrophs **(B)** with soil properties. Colors represent the *rho-value* of Spearman correlations between the relative abundances of taxonomic groups and soil properties. The numbers in the right indicate the relative abundance of methanogens and methanotrophs in hollow (blue) and hummock soils (red; mean ± SE). Asterisks in the box indicate significant differences in the relative abundance between hollow and hummock soils (^***^p < 0.001, ^**^p < 0.01, and ^*^p < 0.05).

In the present study, a total of 15 *mcrA* genes OTUs were obtained and classified. Within these OTUs, three were affiliated with the *Methanomassiliicoccales* (Figure 4A), which together accounted for about 50% of the sequences retained (Figure 4C). Four additional OTUs were affiliated with *Methanomicrobiales* and accounted for another 25%. The other OTUs were affiliated with the *Methanobacteriales* (three OTUs), *Methanosarcinales* (two OTUs), *Methanotrichales* (two OTUs), and *Methanocellales* (one OTUs), which accounted for the remaining 25% of the community. No significant differences were identified between hollow and hummock soils in *Methanobacteriales*, *Methanocellales*, *Methanomicrobiales*, and *Methanotrichales* (Kruskal–Wallis test, *p* = 0.413, 0.269, 0.075, and 0.962, respectively; Supplementary Figure S2A). The relative abundances of *Methanomassiliicoccales* and *Methanosarcinales* were found to be more abundant in hummock soil samples than in hollow soil samples (*p* = 0.005 and *p* < 0.001, respectively). Furthermore, the relative abundance of three OTUs (OTUs 1, 3, and 9) was significantly higher in the hummock soils, and their relative abundance all exhibited negative correlations with pH (Figure 5A). In comparison, the relative abundance of eight OTUs was significantly higher in the hollow soils (OTUs 4, 6, 7, 8, 11, 12, 14, and 15), and their relative abundance all exhibited positive correlations with pH value.

Taxonomic analysis of *pmoA* genes revealed the dominance of type I methanotrophs (Figure 4B). Type Ia methanotroph *Methylobacter* was the most abundant, accounting for approximately 42% of the sequences retained (Figure 4D). Other type Ia (LP20, *Methylomonas* and Aquifer-cluster) and type Ib group (FWs) were also identified but with much lower abundance. In addition, type II methanotrophs (*Methylocystis*) were detected with an average relative abundance of 21.6%. Hollow soil samples exhibited a significantly higher relative abundance of *Methylobacter* (Kruskal–Wallis test, *p* = 0.010) and *Methylomonas* (*p* < 0.001) than that in hummock soils, while a higher relative abundance of *Methylocystis* (*p* = 0.013) and FWs (*p* = 0.017) were detected in hummock soils (Supplementary Figure S2B). The relative abundance of *Methylobacter* significantly correlated with soil water content and organic matter negatively (*p* = 0.012 and *p* = 0.040, respectively), while the relative abundance of *Methylomonas* significantly correlated with pH and available phosphorous positively and negatively, respectively (*p* < 0.001 and *p* = 0.005, respectively; Figure 5B). The relative abundance of *Methylocystis* positively correlated with soil moisture (*p* = 0.008), while FWs negatively correlated with pH (*p* = 0.036) and positively correlated with available phosphorous (*p* = 0.005).

## Discussion

### Glacier Foreland Soil as an Atmospheric CH_4_ Source During the Melting Season

The copy number of the *mcrA* gene was typically higher than that of the *pmoA* gene in the glacier melting season (Figure 2A). Although the qPCR result does not reflect the actual number and activity of microorganisms, the *mcrA*-to-*pmoA* gene copy number ratio has commonly been used to estimate the relative abundance between methanogens and methanotrophs (Morris et al., 2016; Yuan et al., 2018; Kong et al., 2019). Thus, our results suggest that the methanogens were dominant over methanotrophs in the glacier melting season, and the LXZ glacier foreland soils could be a methane source. This was confirmed by the positive *in situ* methane flux, which showed that glacial meltwater turned glacier foreland meadow soils from a methane sink to a methane source (Figure 2B).

The LXZ glacier foreland meadow was a weak methane sink during the pre-melting season (−3.76 ± 9.84 μg·m^−2^·h^−1^; Figure 2B). However, its methane oxidation capacity was much smaller compared with other alpine meadows, which typically ranged from 28 to 71.5 μg·m^−2^·h^−1^ (Lin et al., 2009; Wei et al., 2015b; Wu et al., 2020). Glacier meltwater turned glacier foreland soils into a methane source. This also differed from other glacier foreland soils, which are predominately identified as atmospheric methane sinks (or as weak methane sources) during the glacier melting season, such as observed in Greenland (−0.76 ~ −0.14 μg·m^−2^·h^−1^; Bárcena et al., 2010), Switzerland (Damma and Griessfirn; −2,200 ~ −82 μg·m^−2^·h^−1^; Chiri et al., 2015, 2017), and Svalbard (−110 ~ 300 μg·m^−2^·h^−1^; Adachi et al., 2006). These contrastive differences could be attributed to the soil water content difference, which is a major driver of methane flux (Le Mer and Roger, 2001; Xu et al., 2010). The soil water content of the glacier foreland soils in the Arctic and the Alps (Chiri et al., 2015; Hofmann et al., 2016) is typically less than 30% even during the melting season. This is much lower compared with the soil water content in the present study (>72%), which could be due to the rapid glacier melting on the Tibetan Plateau (Yao et al., 2004; Qiu, 2008). Thus, the enhanced glacial meltwater discharge increased soil water content, which subsequently changed the redox potential and favored methanogens over methanotrophs (Hofmann et al., 2013).

Our results revealed a hidden methane source associated with annual glacial melting on the Tibetan Plateau. This seems to be unique compared with the glacier foreland soils in the Arctic and the Alps (Adachi et al., 2006; Bárcena et al., 2010; Chiri et al., 2017). This uniqueness could be associated with the faster glacier mass loss and greater glacial meltwater discharge (Adachi et al., 2006; Chiri et al., 2015, 2017), as the glacier mass change rate of the TP is faster than those in the Alps and Svalbard regions (Wouters et al., 2019). The Tibetan Plateau has the largest number of mid-latitude glaciers, thus the impact of glacier melting on the glacier foreland methane emission could be overlooked, which needs to be carefully evaluated in future studies.

### Abundance and Diversity of Methanogenic and Methanotrophic Communities

The copy numbers of marker genes (*mcrA*) for methanogens in LXZ glacier foreland soils ranged from 1.19 × 10^6^ to 5.17 × 10^8^ copies g^−1^ dry soil, which is higher than those in the foreland soils of the Alps (4.6 × 10^4^–2.5 × 10^6^ copies g^−1^ dry soil; Hofmann et al., 2016) and the meadow soils of the Tibetan Plateau (6.0 × 10^5^–6.7 × 10^6^ copies g^−1^ dry soils; Yang et al., 2017). The *mcrA* gene copy number increased with elevated soil water content (Figure 3), which is consistent with the results in rice paddy fields (Ma et al., 2012) and Arctic soils (Høj et al., 2006). This finding indicates that the increase in soil water content can enhance the abundance of methanogens, and subsequently increase methane production.

Our results showed that the methanogens in LXZ glacier foreland soils were dominated by *Methanomassiliicoccales* (49%) and *Methanomicrobiales* (24%), which are consistent with those recovered from the NamCo wetland (Deng et al., 2019), Zoige wetland (Zhang et al., 2008), and Qilian Mountain alpine permafrost soils (Wang et al., 2020). *Methanomassiliicoccales* has been identified from a wide range of environments, such as tropical peat swamp forest soils (Too et al., 2018), lake sediment (Emerson et al., 2020), and alpine cave soils (Jurado et al., 2020). *Methanomassiliicoccales* is phylogenetically distant from other methanogen orders, and belongs to a large evolutionary branch composed of many non-methanogenic archaea (Borrel et al., 2014). The wide distribution of these archaea suggests that they could be adapted to psychrophilic/mesophilic conditions, and their methanogenic activity in LXZ glacier foreland soils may be further enhanced under global warming. In comparison, the other dominant hydrogenotrophic methanogen *Methanomicrobiales* were also dominant in the foreland soils of the Alps, and have also been identified from Antarctica and Alaska marine sediment (Dong and Chen, 2012), indicating their adaptation to the psychrophilic environment (Hofmann et al., 2016).

The *pmoA* gene abundance varied from 4.76 × 10^5^ to 1.09 × 10^8^ copies g^−1^ dry soil, which is higher than the siliceous and calcareous foreland soils in Switzerland (8.2 × 10^2^ to 5.5 × 10^5^ copies g^−1^ soil; Chiri et al., 2015, 2017), foreland soils in Norway (~10^3^ g^−1^ soil; Mateos-Rivera et al., 2018), and foreland soils in the Central Alps (~10^3^–10^5^ g^−1^ dry soil; Hofmann et al., 2016). Type I methanotrophs *Methylobacter* and type II methanotrophs *Methylocystis* dominated the LXZ glacier foreland soils. These lineages have been identified in the wetland soils of the Tibetan Plateau (Deng et al., 2014; Zhang et al., 2019), foreland soils of Griessfirn Glacier (Chiri et al., 2017), and Swiss foreland soils (Nauer et al., 2012). In contrary to our results, USCγ cluster is the dominant type I methanotrophs in upland soil ecosystem, such as those reported in glacier foreland upland soils (Nauer et al., 2012; Chiri et al., 2017), Tibetan upland grassland soils (Deng et al., 2019), and Canadian upland tundra soils (Martineau et al., 2014). Members of the USCγ cluster exhibit high methane uptake affinity, and are capable of atmospheric methane scavenging (Knief et al., 2003). The observed difference in the USCγ cluster could be ascribed to the different environmental conditions, such as soil water content. In general, USCγ is more abundant in regions with low precipitation, whereas low-affinity methanotrophs (such as *Methylobacter*) dominate soils with high water content (Deng et al., 2019). The high-water content favors anaerobic methanogens, subsequently leading to enhanced methane production (Huttunen et al., 2003), which supports the growth of low-affinity methanotrophs (such as *Methylobacter* and *Methylocystis*).

### Methane Flux and Methane-Cycling Microorganisms Within Microtopography

Our results illustrated that the hollow soils were a sink for atmospheric methane in the pre-melting season, while hummock soils were a weak source (Figure 2B). This could be due to the presence of vegetation in hummocks (Laine et al., 2007), as root respiration can enhance methanogenic activity and methane emissions by creating an anaerobic environment and providing nutrients *via* root extrudes (Sutton-Grier and Megonigal, 2011; Minick et al., 2019). Glacial meltwater discharge during glacier melting season made both hollow and hummock soils into methane sources (Figure 2B). This is evidenced by the high soil water content (Figure 2D), which can alter the redox potential by reducing oxygen availability (Wadham et al., 2007; Hofmann et al., 2013).

Our results further demonstrated the distinct compositions of methane-cycling microorganisms in glacier foreland hollow and hummock soils. Hummock soils enriched methanogens that negatively correlated with soil pH, whereas hollow soils enriched methanogens that positively correlated with pH. Our results also illustrated pH was the driving factor that regulates methanogens in glacier foreland hollow and hummock soils (Figure 5A). Hummock soils exhibited lower pH values than the hollow soils, thus suggesting hummock soil-dwelling methanogens could have a lower pH preference than those in hollow soils. This is consistent with a previous study that pH is the primary factor influencing methanogen distribution (Deng et al., 2014). Further to this, certain OTUs within the same order (i.e., *Methanobacteriales* and *Methanomassiliicoccales*) exhibited distinct pH preferences (Figure 5A). This finding may indicate the distinct adaptation strategies of the methanogens in the same order. In contrast, *Methanomicrobiales*, *Methanosarcinales*, and *Methanotrichales* were only enriched in hollow soils. Both *Methanomicrobiales* and *Methanosarcinales* are versatile regarding carbon sources compared with those more specialized species (such as the *Methanobacteriales* and *Methanomassiliicoccales*; Angelidaki et al., 2011). *Methanomicrobiales* and *Methanosarcinales* (Conrad, 2020) are also known to be oxygen tolerant, attributed to the presence of superoxide dismutase, which can protect them from oxygen toxicity (Guerrero-Cruz et al., 2018). This can be particularly important for methanogens in hollow soils, where the soil water content can be much lower and the soils can become aerated during the pre- and post-melting season.

The relative abundance of methanotrophs *Methylobacter* (Ia) and *Methylomonas* (Ia) was higher in hollow soils, while *Methylocystis* (IIa) and FWs (Ib) dominant in hummock soils (Figure 5B). This finding is supported by previous observations derived from arctic peat soils (Tveit et al., 2014), lake sediment (Rahalkar et al., 2007), and peat bog soils (Danilova et al., 2016). *Methylobacter* and *Methylomonas* have been found to present in habitats with very low oxygen concentration. During melting season, glacier foreland soils are flooded by the meltwater, providing a very low oxygen condition that allows the more appropriately adapted *Methylobacter* and *Methylomonas* taxa to grow, dominating in hollow soils. In comparison, Type II methanotrophs such as *Methylocystis* have frequently been identified in plant rhizosphere (Bender and Conrad, 1995), partially due to their ability to fix nitrogen (Mäkipää et al., 2018). Furthermore, *Methylocystis* could grow on multi-carbon compounds such as acetate (Hakobyan and Liesack, 2020), which is available through root exudates (Girkin et al., 2018).

## Conclusion

This study shows that meadow soils in the glacier forefield of a receding glacier are an overlooked methane sink in the non-melting season and can be transferred into a methane source in the melting season, and this transformation is regulated by glacier melting and also influenced by local vegetation coverage. Furthermore, the methane production and oxidation balance can be further changed under the projected global warming and accelerate glacier retreat.

## Data Availability Statement

The datasets presented for this study can be found in the NCBI Sequence Read Archive under study accession number PRJNA679195. The soil properties and methane fluxes in present study have been archived at the PANGAEA dataset (https://issues.pangaea.de/browse/PDI-31033 and https://issues.pangaea.de/browse/PDI-31032).

## Author Contributions

TX, PL, YD, and MJ performed the experiments, analyzed the data, and prepared figures and tables. TX and YD drafted the work. YL, MJ, YD, KL, and PL conceived and designed the experiments and approved the final draft. WW and YL contributed to field work. All authors contributed to the article and approved the submitted version.

## Funding

This research was funded by National Key R&D Program of China (Grant No. 2019YFC1509103), the National Natural Science Foundation of China (Grant Nos. 41971077 and 91851207), the Second Tibetan Plateau Scientific Expedition and Research (STEP) program (Grant No. 2019QZKK0503), and the Strategic Priority Research Program (A) of the Chinese Academy of Sciences (Grant No. XDA20050101).

## Conflict of Interest

The authors declare that the research was conducted in the absence of any commercial or financial relationships that could be construed as a potential conflict of interests.

## Publisher’s Note

All claims expressed in this article are solely those of the authors and do not necessarily represent those of their affiliated organizations, or those of the publisher, the editors and the reviewers. Any product that may be evaluated in this article, or claim that may be made by its manufacturer, is not guaranteed or endorsed by the publisher.

## Footnotes

^1^ http://itol2.embl.de
