# Supplementary material for: Sink or Source: Alternative Roles of Glacier Foreland Meadow Soils in Methane Emission Is Regulated by Glacier Melting on the Tibetan Plateau
Source: Front Microbiol. 2022 Mar 21;13:862242. doi: 10.3389/fmicb.2022.862242 (PMC8977769; doi:10.3389/fmicb.2022.862242)
Supplement: Supplementary file 1 [file Data_Sheet_1.docx]

Supplementary Material

# Supplementary Figures and Tables

##
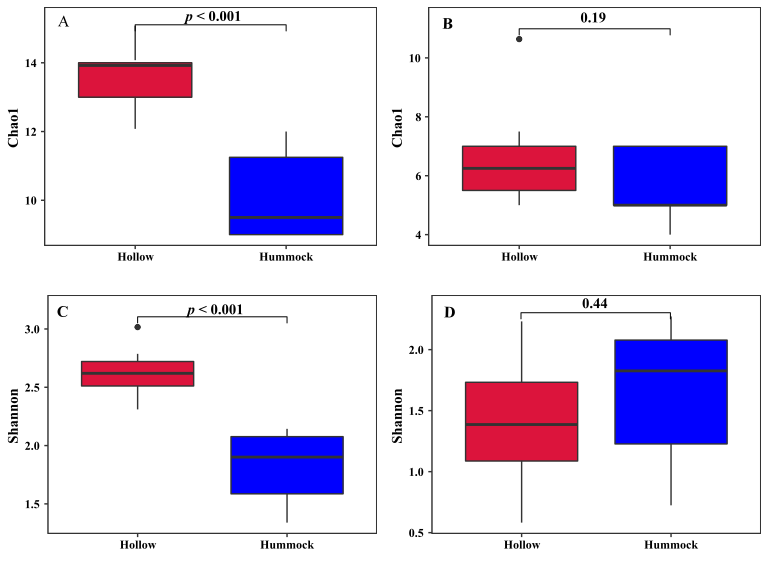
 Supplementary Figures

**Figure S1** Chao1 richness and Shannon index of methanogens (A & C), methanotrophs (B & D) in hollow (red) and hummock soils (blue).


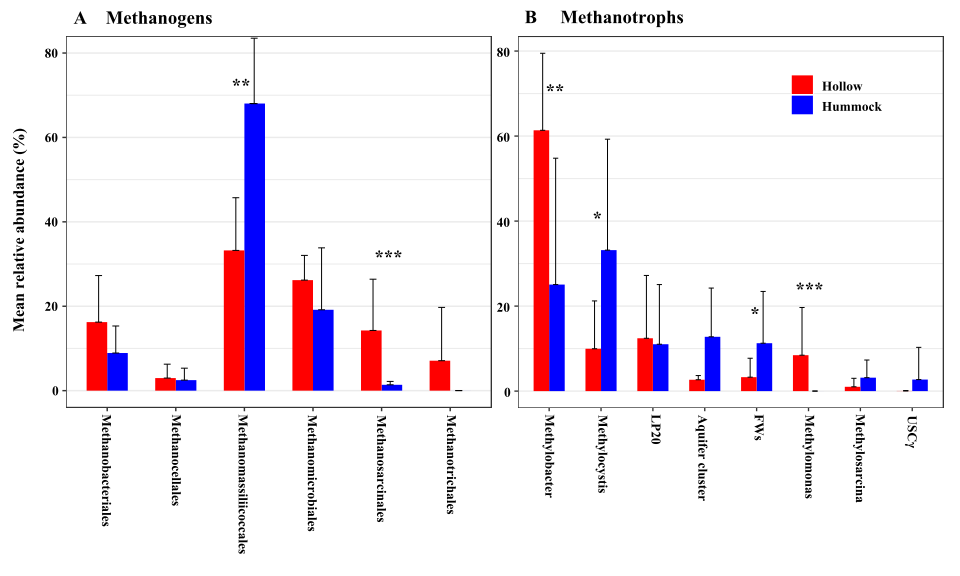
**Figure S2** The mean relative abundance of methanogens (A) and methanotrophs (B) in hollow (red) and hummock (blue) soils. Error bars indicate standard deviation. Asterisks indicate significant difference between hollow and hummock soils (****P* < 0.001, ***P* < 0.01, **P* < 0.05).

**2.1 Supplementary Tables**

**Table S1.** Soil physiochemical properties (mean ± se; n= 3). Hu1-3 in ID mean hummock soils, and Ho1-3 mean hollow soils at site 1 to site 3.

| ID | pH | Moisture (%) | NH_4_^+^ (mg/L) | NO_3_^-^ (mg/L) | OM (g/kg) | TN (g/kg) | AP (mg/kg) |
| --- | --- | --- | --- | --- | --- | --- | --- |
| Ho1 | 7.73±0.15 | 114.27±10.19 | 1.46±0.27 | 0.56±0.18 | 92.28±6.84 | 4.52±0.34 | 15.13±10.62 |
| Ho2 | 7.95±0.14 | 131.42±9.44 | 1.56±0.15 | 0.32±0.04 | 98.61±13.96 | 3.93±0.76 | 9.01±1.56 |
| Ho3 | 7.47±0.08 | 74.81±1.91 | 1.40±0.23 | 0.34±0.07 | 48.18±4.31 | 2.15±0.12 | 6.15±0.71 |
| Hu1 | 7.00±0.07 | 122.42±9.30 | 1.90±0.50 | 0.35±0.06 | 88.12±14.48 | 3.27±0.61 | 17.94±8.21 |
| Hu2 | 7.13±0.05 | 116.85±10.55 | 1.76±0.24 | 0.92±0.17 | 121.63±7.75 | 5.08±0.58 | 16.18±5.11 |
| Hu3 | 7.10±0.06 | 122.42±9.30 | 1.33±0.24 | 0.32±0.03 | 58.33±2.92 | 2.82±0.32 | 14.4±3.74 |

**Notes: NH+ 4**: Ammonium Nitrogen content; **NO- 3**: Nitrate content; **OM**: Soil Organic Matter; **TN**: Total Nitrogen; **AP**: Available Phosphorus content.

**Table S2** *mcrA* and *pmoA* gene abundances in hollow and hummock soils.

| Microtopography | Site | *mcrA g*ene abundance (10^7^ copies g^-1^ dry soil) (mean±se) | *pmoA g*ene abundance (10^7^ copies g^-1^ dry soil) (mean±se) |
| --- | --- | --- | --- |
| Hollow | Ho1 | 0.22 ± 0.08 | 0.09 ± 0.04 |
|  | Ho2 | 31.79 ± 14.47 | 8.04 ± 2.05 |
|  | Ho3 | 10.74 ± 4.00 | 0.57 ± 0.20 |
| Hummock | Hu1 | 1.62 ± 0.77 | 0.54 ± 0.23 |
|  | Hu2 | 0.56± 0.22 | 1.06 ± 0.69 |
|  | Hu3 | 5.99 ± 2.79 | 0.33 ± 0.02 |
